# The Evolutionary Pathway to Obligate Scavenging in *Gyps* Vultures

**DOI:** 10.1371/journal.pone.0024635

**Published:** 2011-09-08

**Authors:** Brian J. Dermody, Colby J. Tanner, Andrew L. Jackson

**Affiliations:** 1 Department of Zoology, School of Natural Sciences, Trinity College, Dublin, Ireland; 2 Department of Environmental Sciences, Copernicus Institute of Sustainable Development, Utrecht, The Netherlands; American Museum of Natural History, United States of America

## Abstract

The evolutionary pathway to obligate scavenging in *Gyps* vultures remains unclear. We propose that communal roosting plays a central role in setting up the information transfer network critical for obligate scavengers in ephemeral environments and that the formation of a flotilla-like foraging group is a likely strategy for foraging *Gyps* vultures. Using a spatial, individual-based, optimisation model we find that the communal roost is critical for establishing the information network that enables information transfer owing to the spatial-concentration of foragers close to the roost. There is also strong selection pressure for grouping behaviour owing to the importance of maintaining network integrity and hence information transfer during foraging. We present a simple mechanism for grouping, common in many animal species, which has the added implication that it negates the requirement for roost-centric information transfer. The formation of a flotilla-like foraging group also improves foraging efficiency through the reduction of overlapping search paths. Finally, we highlight the importance of consideration of information transfer mechanisms in order to maximise the success of vulture reintroduction programmes.

## Introduction

Certain species of *Gyps* vultures represent the only extant vertebrate obligate scavengers on earth, having evolved highly specialised physiologies and behaviour to enable them to exploit spatially rare and temporally ephemeral food resources at the cost of the ability to kill prey [1], [2]. However, the evolutionary pathway to obligate scavenging remains unclear. It is already well established that information transfer among conspecifics is central to foraging success for a range of avian foragers in ephemeral environments [3]–[6]. For instance, a food patch is easier to detect for foragers once it has been discovered owing to increased activity associated with an aggregation of feeders [6]. A potentially more important mechanism for information transfer among vultures occurs by observing the behaviour of conspecifics in the sky. When a vulture discovers a carcass it drops its feet, which increases drag and causes the bird to descend [7]. This action is observed by other vultures and they in turn descend in the direction of the descending bird, creating a chain of descending vultures within visual range. This process is so efficient that it can lead to several hundred birds reaching a carcass within hours of the initial discovery [8]. Given that information transfer is dependent on visual cues from conspecifics, the density of foraging birds in the sky is critical as birds must remain in visual range of at least one other conspecific to access the information transfer network.

We propose therefore, that vultures in foraging flight adjust their speed and direction, within the constraint of soaring flight, to remain in visual contact with conspecifics thus maintaining access to the information network throughout the foraging day. Houston [9] states that foraging vultures are usually within visual contact of one or more conspecifics and New World vultures have been observed setting out on foraging trips in temporally clumped groups, which is proposed as an intermediate step to group foraging [5]. Indeed, when it is considered that hundreds of vultures are recruited to carcasses within an hour of discovery from distances of up to 35 km, it seems likely that they have access to a common information network [2], [8]. In fact, *Gyps* vultures in the Serengeti Ecosystem are thought to find food almost every day they forage [9].

Whilst group foraging is proposed to be important for information transfer, we cannot ignore the role the roost plays in terms of information transfer among vultures. Many avian species that rely on food sources that are large or spatially aggregated but also temporally and spatially ephemeral roost communally [3]. There is little doubt that in ephemeral environments communal roosts improve information transfer among conspecifics and thus reduce variance between feeding bouts as opposed to dispersed roosting [10]. However, the mechanism by which information transfer occurs among communally roosting avian species has been a source of debate for a number of decades [3], [5], [6], [11]–[15]. Much of the discussion surrounding information transfer in communally roosting avian flocks centres on whether roost-centric information transfer mechanisms such as the information centre hypothesis exist, or whether extra-roost mechanisms such as local enhancement offer sufficient explanation for the evolution of communal roosting.

We propose that extra-roost mechanisms are sufficient to impose selection pressure for communal roosting; specifically the fitness benefits vultures derive from being spatially concentrated at the beginning of the foraging day. In addition, we propose that the aggregative properties of the roost are essential for the formation of vulture foraging groups that would otherwise take time to form through coalescence in the wider habitat.

We propose a simple set of rules for dynamic grouping that appear to form the basis of many complex animal groups such as fish shoals, flocks of birds and swarms of insects as a probable strategy for foraging vultures [16]. These simple models require three zones be defined around an animal in increasing order. Animals in these models simply repel each other if they are within the first zone, orientate and align their direction within the second zone, and attract each other within the last and widest zone. Through minor adjustments in the relative radii of these zones, complex group-level patterns emerge that are capable of a variety of collective behaviours such as swarming, toroidal rotation and polarised motion. In animal groups that display dynamic coordinated movement, the primary evolutionary driver is generally regarded as improved information transfer to facilitate resource detection, prey avoidance and so on. This raises the question as to whether roosting or grouping is the primary mechanism for information transfer among communally roosting bird species.

To compare the relative success and hence evolutionary pressure towards communal roosting and active flock formation, we define four strategies and compare their food-finding abilities using simulation models. Each strategy is compared across equivalent environments (defined in Methods section). We compared strategies where vultures begin the foraging day from a common location (roost) or randomly dispersed throughout the habitat (dispersed). In each case vultures either ignore the presence of conspecifics unless descending or feeding (individual) (*sensu* Jackson et al. [17]) or react to conspecifics within their field of perception (group). Henceforth we refer to these foraging strategies as individual-roost, individual-dispersed, group-roost and group-dispersed, respectively.

We use a spatial, individual-based, optimisation model to test our hypotheses with vultures competitively interacting whereby the most efficient foragers are preferentially selected for reproduction according to the principles of natural selection. Therefore, individuals in a simulation evolve according to an individual rather than a Pareto optimum. In this manner we can be confident that we are comparing the optimum individual behaviour across environments given the restriction of the foraging strategy under investigation.

Given that our model is spatial and self-optimising, it is also instructive of the optimum group structure for foragers in ephemeral habitats. Previous modelling studies have indicated that active flock formation is beneficial for foraging vultures [5], [18], however the detailed behavioural mechanisms or group structure were not explicitly defined. In light of our findings we highlight important factors that should be considered for vulture conservation programmes.

## Methods

Simulation models were developed using Netlogo; a multi-agent, individual-based modelling environment [19]. The model space is a 2 dimensional, simulated 66×66 km square with periodic boundary conditions so that a vulture flying out one side of the model will appear seamlessly on the opposite side. Therefore the model can be interpreted as a section of a larger habitat free from ecologically unrealistic edge effects [20]. Each iteration of the model represents 10 seconds in real time. This resolution was deemed adequate as large soaring birds such as vultures are unlikely to change direction more than once in this time period [7]. A foraging day is assumed to last for 3 hours with N vultures setting off in search of M randomly distributed carcasses (Jackson et al. 2008). In all strategy sets. vultures are given a random initial direction and their flight speed is fixed at 33 km h^−1^ [21].

The foraging behaviour of each vulture is determined by its foraging state, which changes relative to the location and state of carcasses and other vultures within its field of vision. The three possible states of an individual at any moment are searching, descending or feeding.

### Searching

Vultures begin each day in searching flight. While searching, vultures travel at constant speed and change direction with individually determined turn rates and turn angles. For grouping vultures, direction is also modified by the heading and distance of other vultures within their field of vision (vultures are assumed to have 360° vision with a range of 4 km) [17]. Grouping vultures perform repulsion, orientation or attraction behaviour dependent on other vultures in their zone of repulsion (ZoR), zone of orientation (ZoO) and zone of attraction (ZoA). Fine-scale manipulation of the extent of these zones enables a suite of complex group behaviours to manifest [16]. For example, if a vulture's ZoR, ZoO and ZoA are set to 1, 2 and 4 km respectively the vulture will move away from vultures within a 1 km radius of itself, align with vultures in a 1–2 km radius and move towards vultures within a 2–4 km radius. The maximum angle through which a vulture can turn in response to conspecifics is constrained to the difference between its heading and the mean heading of all vultures within visual range. Therefore, ecologically improbable behaviour such turning through 180 degrees in a single iteration is avoided [22].

### Descending

When a vulture encounters a carcass its state changes and it begins descending. If other vultures are within visual range of a descending bird, they follow the closer vulture in descent to the carcass. This mechanism of social information transfer enables a vulture to locate a carcass without seeing it directly. Depending on spatial dispersal, chains of descending vultures can develop with each member of this chain following the closest descending vulture within a 4 km radius. The following vulture flies towards its leader until it detects the carcass for itself, whereupon it adjusts its heading to intercept the carcass directly [17].

### Feeding

Once a vulture reaches a carcass it remains stationary, its state changes to ‘feeding’, and its time spent at the carcass is recorded. The carcass state is changed from ‘unoccupied’ to ‘occupied’ and the carcass detection range changes from 0.3 km to 4 km to represent that carcasses are detectable from a greater distance with the aggregation of feeding vultures.

### Optimisation

The values for the five parameters that control vulture flight (turn rate, turn angle, ZoR, ZoO and ZoA) are randomly generated for each vulture at the beginning of the simulation. To find optimal values for these parameters, a genetic algorithm is applied to the initial population whereby 10% of individuals, (20% for simulations where N  =  5) with the most effective foraging strategies (measured by proportion of time spent feeding) at the end of each generation are preferentially selected for reproduction through a process known as elitist selection [23]. In the optimisation process each parameter is represented by a gene, with the combination of these genes determining the phenotype or behaviour of each bird. Reproduction is asexual and haploid, with a 0.02 and 0.005 probability of genetic mutation for populations of ≤20 and >20 respectively. A greater probability of mutation for smaller populations is necessary to maintain genetic diversity and achieve optimisation [23]. The simulation is run for 100 generations, with each generation comprising 100 foraging days. The genetic algorithm serves to evolve behavioural phenotypes that represent locally optimal strategies for individuals seeking to maximise their energetic intake. Thus, we have not optimised the flock as a single unit, but rather allow selection to act on the individual. It is important to note that we are not interested in the resultant genotypes *per se* (i.e. we do not consider the evolved parameter values themselves), but rather focus our attention on the performance of the resultant phenotype (i.e. what behaviour manifests at the individual and group level).

### Foraging simulations

Simulations are performed for group-roost, individual-roost, group-dispersed and individual-dispersed foraging strategies, and optimised behaviours in each strategy set are then compared. Roosting vultures are randomly distributed within a 10×10 km square at the beginning of each foraging day to reflect the situation a short time after leaving a roost site on a foraging trip. Dispersed vultures are randomly distributed throughout the entire model space at the beginning of each foraging day. Vulture numbers and carcass numbers are varied among simulations to investigate the respective impacts of forager density and carcass density on foraging efficiency. Final fitness values are determined as the mean fitness (time feeding) of the final 20 generations of the simulation [23]. Where vulture numbers are kept constant and carcass density varied, we analysed the response of the 4 foraging strategies using a generalised linear mixed-effects model, treating foraging type as a fixed effect and carcass number as a random effect [24]. We conducted a similar analysis to compare foraging strategies across a range of vulture densities with carcass density fixed and vulture density treated as a random effect.

## Results

**Figure 1:**
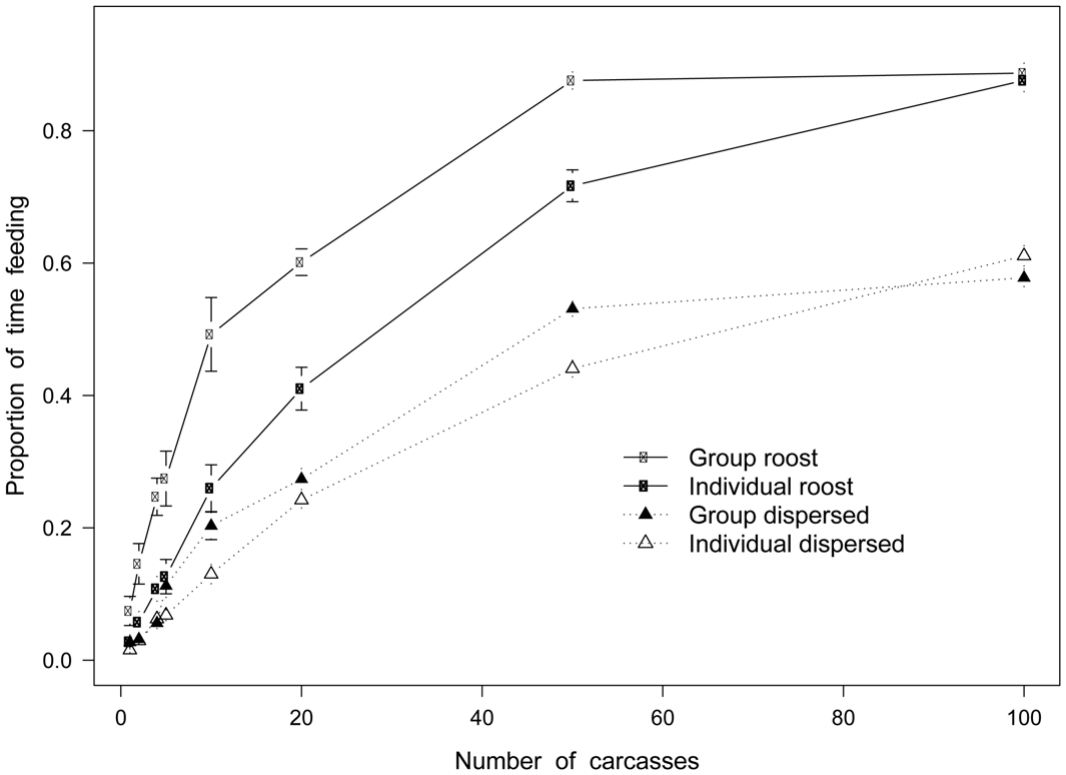
Fitness under alternating carcass density. Proportion of time feeding (±1SD) for 20 simulation replicates carried out across increasing carcass numbers for 4 foraging strategies (n = 20 vultures each simulation run).

We examined the impact that variation in carcass density has on the foraging efficiency of a population of 20 vultures for each strategy under investigation: group-roost, individual-roost, group-dispersed and individual-dispersed (Figure 1). Across all strategies, fitness increases asymptotically with increasing carcass density. As expected, group-roost represents the optimum strategy except at very high carcass densities where it is equivalent to individual-roost. Otherwise, roost and group strategies for the most part outperform dispersed and individual strategies for all carcass densities.

**Figure 2:**
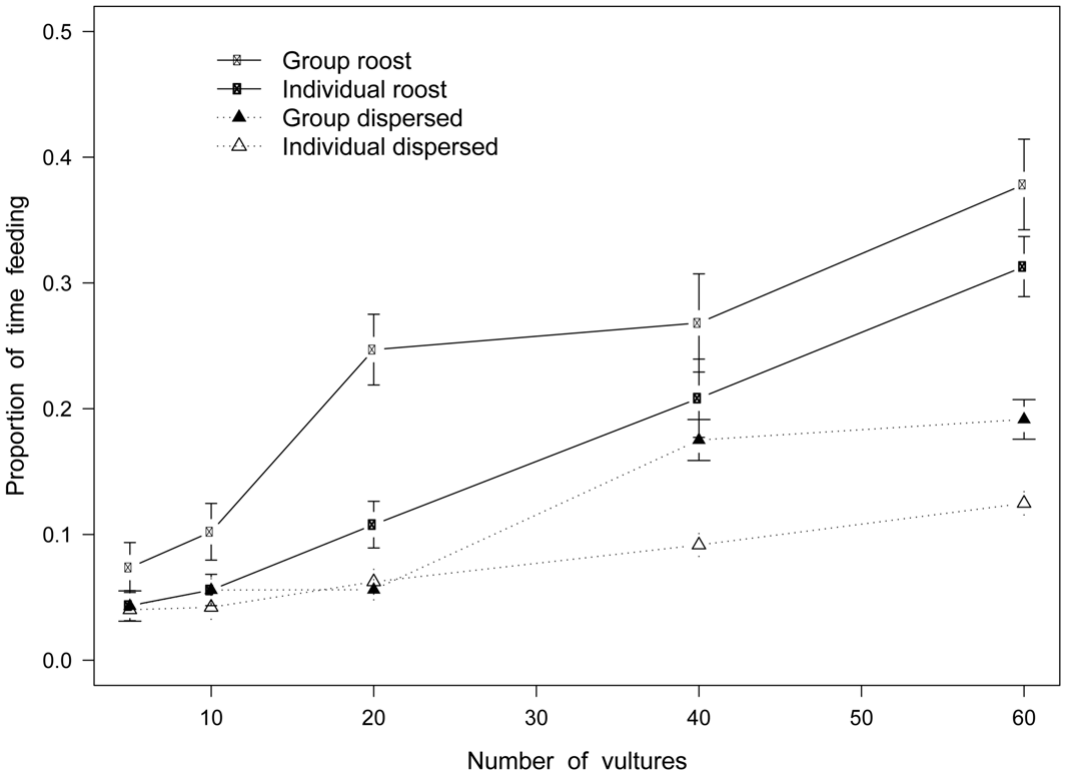
Fitness under alternating vulture density. Proportion of time feeding (±1SD) for 20 simulation replicates carried out across increasing vulture numbers for 4 foraging strategies (carcass density = 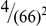 Km each simulation run).

We also examined how changes in forager numbers affect foraging efficiency under the same 4 strategies with carcass density kept constant at 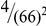 Km: the average carcass density in the Serengeti [25] (Figure 2). Again the group-roost strategy performs best, followed by individual-roost, group-dispersed and individual-dispersed. For all strategies there is an increase in fitness with increasing vulture density, but it is irregular for the group strategies. The largest increase occurs in the group-roost strategy between a population of 10 and 20 birds, where we observe a sharp increase in the ability of birds to locate carcasses. Foraging efficiency also exhibits a sharp increase between a population of 40 and 60 birds under the group-roost strategy. The group-dispersed strategy also displays a sharp increase in foraging efficiency between a population of 20 and 40 birds. In contrast, individual strategies exhibit a linear increase in fitness with increasing vulture numbers.

Taking simulations for varying carcass and vulture densities separately, the group-roost strategy represents the most efficient foraging type (p<0.001 compared with all other strategies in the model).

## Discussion

The evolutionary pathway to obligate scavenging among *Gyps* vultures was explored using individual-based simulation models within the context of a genetic algorithm. We compared the relative fitness of four behavioural strategies each optimised to perform under different ecological scenarios. Although active group formation in combination with communal roosting out-performed all strategies as expected, it is clear that communal roosting provides a more important benefit than active group formation. Such a finding strongly suggests that simple proximity to other vultures at the start of the foraging day is sufficient to set up the information network required to allow vultures locate food. Indeed the importance of spatial concentration was noted previously from empirical observations of New World Black Vultures [5], [6]. The higher selection pressure for communal roosting as opposed to grouping is particularly interesting given that in other animal species where grouping is seen (such as shoals of fish, swarms of insects etc.) the primary conduit for information transfer derives from the maintenance of group cohesion [26]. However, among vultures and other communally roosting birds it appears that large conspecific detection distances mean that the spatial concentration provided by communal roosting is sufficient to provide access to the information network.

Nonetheless there is strong selection pressure for active group formation, with grouping strategies outperforming individual strategies across most simulations except those with very high resource density or very low forager density. A selection for grouping is consistent with previous models of juvenile raven foraging, which found that group foraging is an evolutionarily stable strategy (ESS) in open habitats that are relatively productive and can be searched by the group in a single foraging day [27]. In addition to the ‘many eyes’ advantage that group foraging provides individuals, grouping among juvenile ravens has also been proposed as a mechanism to achieve dominance over adult pairs at a food resource [15], [27]. Further studies of how such resource-centric interactions vary among species [28], [29], especially with changes to group size, will provide important information in understanding the efficiency of group foraging. However, irrespective of such resource-centric interactions, the fact that grouping is selected for in separate species that forage under similar conditions indicates it is a likely strategy among a range of communally roosting avian species that forage in open habitats where resources are large and ephemeral.

Of note in our simulations are the jumps in fitness exhibited for grouping strategies as the number of vultures increases in a constant carcass habitat. This is in contrast to the rather predictable functional forms of fitness in simulations where the numbers of carcasses increases under constant vulture density (sensu Jackson et al. [17]). Given that roost foragers are initially spatially concentrated within a 10×10 km square, essentially, they are randomly dispersed in a smaller area than dispersed foragers (60×60 km square). Among populations of ≤10, density is low and individuals are non-uniformly distributed so smaller, fractured foraging groups form [30]. Where recruitment occurs, it is restricted to members of the subgroup. However, where larger groups forage from a roost, density is such that a unified group consistently forms. The unified group covers a larger search area and where recruitment occurs, all members access the information network. The same pattern can be seen for dispersed populations of 40 vultures as there is sufficient density for vultures to consistently be within visual range of conspecifics and thus form groups, albeit fractured. Therefore, the switching points illustrated here are a result of the initial density of foragers and illustrates that an optimum foraging density exists. Given that Houston and Ruxton [9] state that vultures find food on almost every day they forage, it suggests that soon after leaving the roost vultures form a flotilla-like group rather than forage individually or form fractured subgroups, and thus maintain access to a common information network. Individual foraging at a large enough forager density would also result in every bird accessing the information network, but only for unrealistically high forager densities [17].

Under grouping strategies, the optimum evolved strategy is consistently a highly cohesive, dynamic parallel group. Parallel grouping is common in nature where groups are large; particularly among fish and bird species where shoals can comprise thousands and even millions of members [31]. In these cases parallel grouping is suggested as a method of information transfer through deviation of direction and for energy saving [32]. However, aerodynamics are not included in our model and deviation of direction is not a contributing factor because descending flight is the primary mechanism for information transfer. Hence, the evolution of parallel movement here seems a strategy to minimise overlap of search areas. Therefore, where dynamic parallel groups are seen among foragers in ephemeral environments, consideration should also be given to the role this parallel movement plays in minimising overlap. We do not suggest that such highly parallel movement is quantitatively accurate for vulture foraging groups given the nature of soaring flight and its dependence on accessing thermals and thermal streets [33]. However, the overall features of maintenance of spatial-concentration and reduction of overlap are qualitatively appropriate.

We propose that the roost-centric information transfer mechanisms such as the information centre hypothesis are implausible for *Gyps* vultures given that they generally only return to the roost at the end of the foraging day [9]. Nonetheless, a mechanism for information transfer from knowledgeable to naïve individuals is desirable, particularly in migratory ecosystems where food is non-uniformly distributed [9]. Couzin et al. [32] demonstrated that large animal groups operating under the simple behavioural rules (*sensu* Couzin et al. [16]) applied here can be efficiently led to a resource by a small subset of knowledgeable individuals. The recruitment of conspecifics in this way does not require the recognition of explicit signals or signs of dominance [34] such as body size which is important over the distances at which information transfer occurs among vultures. Simply by maintaining grouping behaviour, uninformed individuals are recruited by informed individuals with a preference of direction. This suggests that where vultures form foraging groups departing from a common roost; information transfer mechanisms at the roost are not required for the transfer of information from knowledgeable to naïve birds.

### Conclusion

Rather surprisingly our results demonstrate that the spatial-concentration of foraging vultures that arises from communal roosting provides a far greater fitness benefit than the maintenance of cohesion associated with grouping behaviour. Nonetheless, there is strong selection pressure for grouping. Under the simple set of rules suggested for grouping among a range of animal species; extra-roost mechanisms offer a sufficient explanation for the types of information transfer observed among *Gyps* vultures and potentially other species of communally roosting avian foragers.

Our findings emphasise that vulture foraging efficiency is density dependent, and thus sensitive to Allee-type effects. Therefore, a reduction in forager density can perturb information transfer mechanisms to the extent that information transfer breaks down entirely and the population accelerates towards extinction [17], [35]. Crucially, the model highlights that vulture foraging success is vastly improved with the inclusion of a communal roost site. This is critical in a conservation context as it demonstrates that reintroduction programmes such as those taking place across the Indian sub-continent [36]–[38] and which will be required in Africa [39] must concentrate their energies on reintroducing large numbers of birds to a few sites with suitable conditions for communal roosting. Such tactics may seem like placing all of one's eggs in the same basket, but the alternative, although superficially more conservative, is potentially disastrous given vultures reliance on density dependent, information networks.
